# Study on Flexural Behaviour of Ferrocement Composites Reinforced with Polypropylene Warp Knitted Fabric

**DOI:** 10.3390/polym14194093

**Published:** 2022-09-29

**Authors:** Manickam Rameshkumar, Ramalingam Malathy, Priyalatha Chandiran, Sundararajan Paramasivam, Ill-Min Chung, Seung-Hyun Kim, Mayakrishnan Prabakaran

**Affiliations:** 1 Department of Fashion Technology, Sona College of Technology, Salem 636 005, Tamil Nadu, India; 2 Department of Civil Engineering, Sona College of Technology, Salem 636 005, Tamil Nadu, India; 3 Department of Handlooms and Textiles, Collectorate Campus, Salem 636 001, Tamil Nadu, India; 4 Department of Crop Science, College of Sanghuh Life Science, Konkuk University, Seoul 05029, Korea

**Keywords:** chicken mesh, composites, ferrocement, flexural strength, polypropylene, warp knitted, SEM-EDX

## Abstract

Ferrocement is a cost-effective construction material used in the low-cost constructions. It is produced with the combination of cement mortar with closely spaced wire mesh known as chicken wire mesh. Ferrocement process eliminates coarse aggregates when compared to reinforced concrete thus makes the process simple. This paper deals with the influence of various characteristics of warp knitted fabric on the flexural properties of ferrocement composites. Ferrocement composites have a wide range of applications in the construction industry and it has some limitations due to the durability issues. Among the various durability issues, corrosion is one of the main issues to be addressed to enhance the long-term service life of the ferrocement composites. The idea of using non-metallic mesh to eliminate the corrosion problem is discussed in this paper. In this experiment, warp knitted fabric reinforced ferrocement composites were produced using polypropylene warp knitted fabrics. This paper deals with the flexural properties of ferrocement composites made of warp knitted fabric coated with expoxy. This paper deals with the flexural properties of ferrocement composites made of warp knitted fabric coated with expoxy. These composites were analyzed for their flexural strength, energy absorption and ductile property. The variables in the experiment are filament thickness, warp knitted structure and number of layers in the composites. Experimental results proved that the replacement of chicken mesh wire by warp knitted fabrics has an impact in the flexural properties of the composites and the effect of variables in the experiment set up has been analyzed. There is an imporvement of 200% is observed in the first crack load and 120% improvement in the ultimate load of the warp knit fabric reinforced composite compared to control sample. Experimental results proved that there is an increase in flexural strength of ferrocement composites made up with warp knitted fabrics. Microstructure studies like SEM and EDX on ferrocement laminates confirmed good bonding between the mortar mix and warp knitted fabrics.

## 1. Introduction

Ferrocement is a special innovative construction material made up of cement mortar with closely spaced wire mesh as a reinforcement. The ferrocement elements can be constructed in any new shape since no course aggregate is used and the meshes can be wrapped to the required shape and mounted in the skeletal rod It can be casted into any shape as it doesn’t use coarse aggregate and steel rods for reinforcement [1,2]. The bonding forces between the chicken mesh and cement mortar are very strong due to high specific surface area in ferrocement [3,4]. Also, the closely spaced wire mesh provides higher ductility to the ferrocement composite and act as a crack arresting element [5,6]. Some of the applications of ferrocement composites are low-capacity water tanks, roof top slabs, thin walls, staircase slabs, toilet elements, park benches, home furniture, book racks, water boats, water troughs and soil erosion prevention structures [7]. Ferrocement composites are extensively used for strengthening of old and deteriorated concrete structures [8,9]. Ferrocement composites are cost effective than the reinforced concrete which is often used in the construction industry. Salgia et. al. [10] studied the expenditure incurred in the ferrocement against reinforced concrete for various constructions and found that the constructions with ferrocement composites are cheaper. The ferrocement composites have many positive features whereas it possess certain limitations. ACI defined durability as the ability to resist action of weather, abrasion and chemical or any other sources which deteriorates the concrete. Similarly, ferrocement also fit into this durability definition [11,12]. As ferrocement composites are used in low-cost constructions, durability is a big question to be addressed in the construction industry. Ferrocement durability is affected by various factors such as less contribution of mortar in mesh reinforcement, low cross-sectional area of wire mesh, zinc coating in the mesh leads to corrosion etc. [13].

The limitations of the ferrocement composites need to be addressed towards which several research works were carried out and also in progress to enhance the performance property, service life and durability of the ferrocement composites [14]. Before identifying the solution to the corrosion resistance, some researchers analyzed the deterioration of ferrocement performance properties by placing the ferrocement composites in the corrosion induced environment. The experimental results proved that the corrosion environment had an adverse effect in the mechanical and flexural properties of the ferrocement composites [15,16]. The durability of the ferrocement composites were studied under normal, medium and hostile environment. In this experiment, woven and hexagonal steel wire mesh was used for reinforcement. Fly ash admixture was added in the cement mortar which enhances the flexural strength properties of the ferrocement composites [17,18]. In the ferrocement casting process, chromium tri oxide admixture is added in the corrosion prevention process. The experimental results proved that the admixture improves the corrosion resistance of ferrocement composites [19,20]. In the ferrocement composites, an attempt has been made to study the effect of laminate thickness, water/cement, sand/cement ratio, content of admixtures in the composite and chicken mesh on the corrosion of the ferrocement composites. In the experiment, the corrosion is measured in terms of loss of steel and 7.9% loss of steel was observed in 25 days from onset of cracking. The corrosion problem ultimately affects the flexural properties of the ferrocement composites [21,22]. Among the various admixtures, fly ash is a popular one used by various researchers to assess the improvement in durability. Fly ash and polymer admixture was used in the mortar mix and performance properties were assessed. Experimental results supported the addition of admixtures in the improvement of corrosion resistance [23,24,25,26,27]. Similar to fly ash and polymer, silica fume is also used as admixture in the ferrocement casting process. This silica fume addition in mortar mix improved the corrosion resistance in composites [28,29,30,31].

Next to the admixtures, some researchers modified the type of cement used in the ferrocement casting process. Two types of cements namely ordinary Portland cement and Portland slag cement is used in the ferrocement casting process. Experimental results proved that Portland slag cement outperformed the ordinary Portland cement in terms of performance properties and durability [32,33]. Apart from the admixtures, attempt has been made to provide polymer coating over the mesh to improve the durability. In an experiment, PVC coated steel mesh has been used in the ferrocement casting process. Experimental results proved an improvement in corrosion resistance in the ferrocement composites [34].

Along with fly ash, polymer and silica fume admixture, sodium nitrite based chemical admixture also tested in the ferrocement composite to improve the corrosion resistance. The results proved this chemical admixture as an effective inhibitor for the corrosion [35]. The modification of mesh variety was implemented by the researchers to improve the durability. The steel wire mesh has been replaced by galvanized wire mesh and dense mortar was prepared using fly ash and silica fume admixture in the experiment. These modifications helped to improve the durability of the ferrocement composites [36,37,38]. But these suggested ways are studied and proved to be ineffective after a certain period of time which reduces the performance properties of ferrocement composites [39,40]. Several alternatives were used by researchers to overcome the limitations of the ferrocement wherein polypropylene warp knitted fabrics were used as a replacement to chicken mesh wire in this experiment. The flexural properties of warp knitted fabric reinforced ferrocement composites were analysed. So it is necessary to identify new material for ferrocement composites to overcome the drawbacks such as corrosion, durability, cost effective and deterioration of structure. The advantages of using polypropylene fabrics to reinforce the ferrocement leads to less corrosion, increase durability and less deterioration of the structure. The waste materials can be reuse for making ferrocement composites.

## 2. Materials and Methods

### 2.1. Matrix and Reinforcment

The objective of the experiment is to assess the flexural properties of the polypropylene warp knitted fabric reinforced ferrocement composites and to compare it with the conventional chicken mesh ferrocement composites. In the process of attaining this objective, the first step is to produce warp knitted fabrics from polypropylene (PP) multi filament yarn. Three different linear densities 93, 187 and 280 tex was used in the experiment in order to study the effect of filament thickness on the flexural properties.

**Figure 1:**
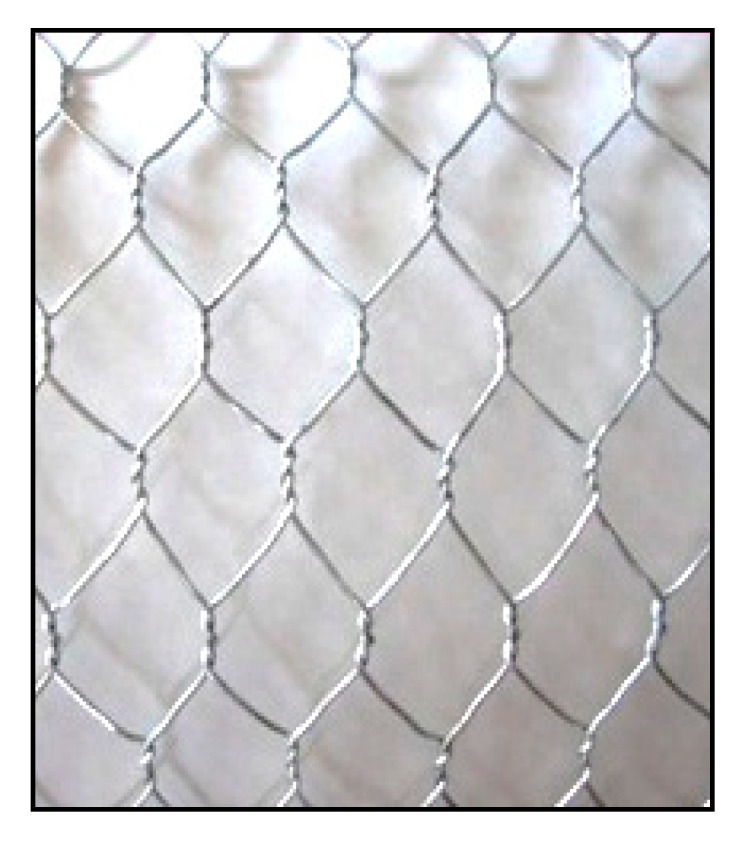
Chicken mesh.

**Figure 2:**
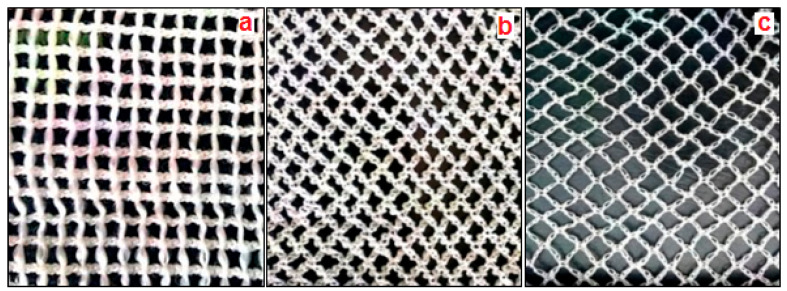
(**a**) Marquisette net 5 mm mesh, (**b**) sandfly net small mesh 5 mm mesh, and (**c**) sandfly net big mesh 10 mm mesh.

Warp knitted samples were produced with three different structures namely marquisette net, sandfly net structure with small and big size mesh. The shape of the mesh in the marquisette and sandfly net is square and diamond shape respectively. Warp knitted samples were produced on locally available 12 gauge warp knitting machine, Robaczynski Corporation, Brooklyn, USA with a working speed of 100 rpm. The size of the mesh in the structure is 5 × 5 mm for marquisette net and sandly small net, 10 × 10 mm for the sandfly big net structure. Figure 1 and Figure 2a–c, represent the chicken mesh and polypropylene warp knitted samples of three different structures. The number of variables of the experiment in the warp knitted fabric production process is six i.e., three different deniers and three different structure. Hence a total of nine warp knitted samples were produced in the warp knitting machine. Table 1 and Table 2 represent the physical properties of cement and fine aggregate. Table 3 represents the specifications of chicken mesh used in the reinforcement of control sample.

#### 2.1.1. Testing of Warp Knitted Samples

**Figure 3:**
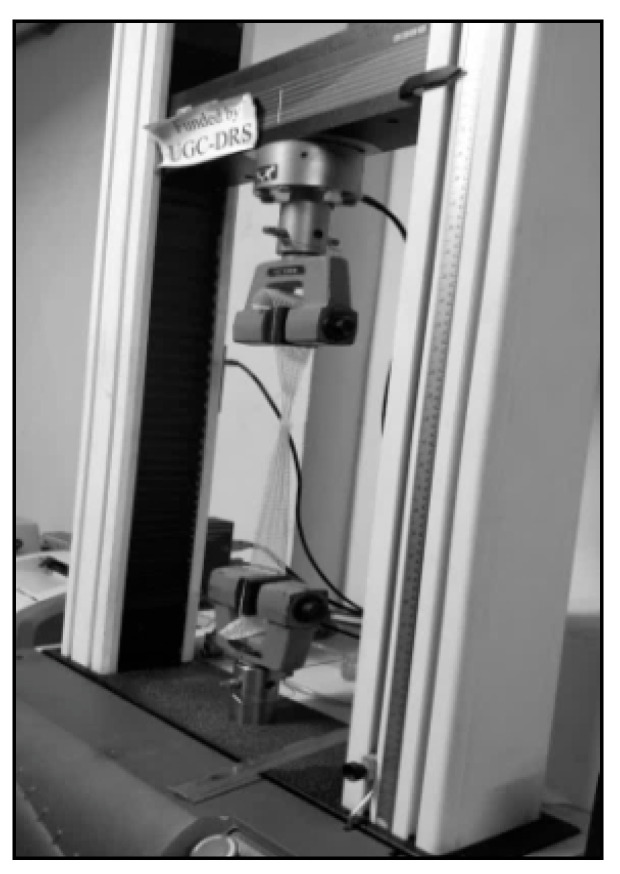
Tensile testing of warp knitted samples.

The entire nine warp knitted samples were tested for tensile properties in Instron Testing instrument. The samples were tested with a gauge length of 200 mm, base length of 50 mm and loading rate is 300 mm/min and the test standard followed in the experiment is ASTM 5034-09 (2017). Tensile stress, tensile strain and young’s modulus properties were measured for the warp knitted samples. Figure 3 represents the warp knitted sample testing process.

#### 2.1.2. Ferrocement Composite Preparation Process

**Figure 4:**
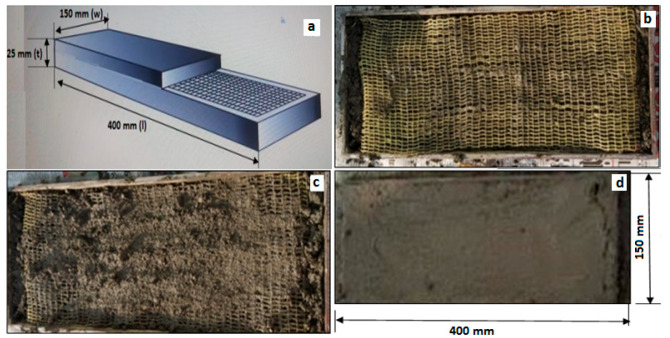
(**a**) Warp knitted fabric with scalebar, (**b**) Warp knitted fabric in mould, (**c**) composite preparation, and (**d**) ferrocement composite casting process.

The components of cement mortar mixture used in the casting process are water, sand and cement. The ratio of water to cement is 1:0.38 and cement to sand is 1:2 in the cement mortar preparation. The type of cement and sand used in the mortar preparation is ordinary Portland cement and river sand respectively. An iron mould is prepared for ferrocement casting with dimensions of 400 mm length × 150 mm width × 25 mm thickness. Warp knitted fabrics were coated with epoxy resin to position the fabric in the mould. Figure 4a–d represents the ferrocement composites preparation process.

In the casting process, initially grease is applied on the inner wall of the iron mould for easy removal of composites from the mould. The prepared mortar mix is placed inside the mould for one layer and then a single layer of fabric is placed in the mould and it is ensured that there is a 5 mm gap between the warp knitted fabric and the inner wall of the mould in order to improve the covering area of mortar mix and to improve the fabric cement bonding. At this point of casting process, a variable has been introduced in the experiment namely number of fabric layers in the composite. Ferrocement composites are prepared with 2, 3 and 4 layers of fabric in the mould. There is a mortar mix layer placed between each fabric layer for good penetration of cement mortar and better fabric cement bonding. After casting, the mould is placed in open atmosphere for one hour and the composites were removed from the mould. The ferrocement composites were placed in the water tank for 28 days for curing process. Table 4 represents the details of the marquisette net, small sandfly net and big sandfly net warp knitted fabric reinforced ferrocement composites sample code used in the experiment with its appropriate description details.

Table 5 Represents the sample code of chicken mesh reinforced ferrocement composites.

#### 2.1.3. Flexural Test Set up for Ferrocement Composites

**Figure 5:**
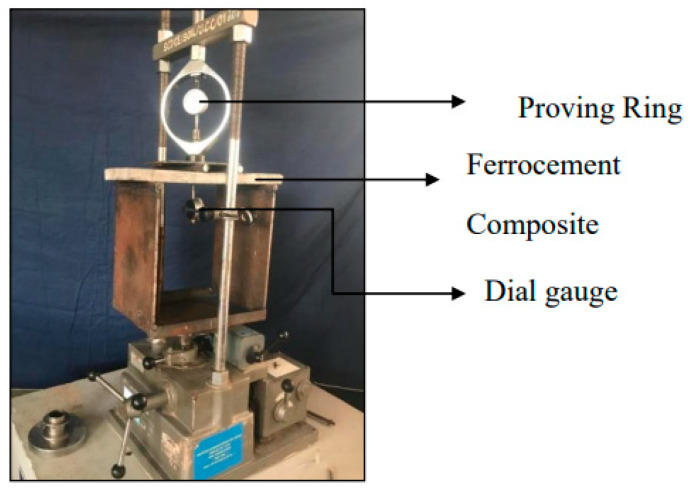
Flexural test set up.

After curing process, the ferrocement composites were dried in the sun light for one day for complete water draining from the laminate and then taken for flexural testing process. In the flexural testing, first crack load, ultimate load, load deflection behaviour, energy absorption capacity and ductility factor were determined for every laminate. The composites were tested for two point load in a universal testing machine. The load is applied at an increment of 0.5 kN and load is indicated in the proving ring at the top of the flexural test set up. For every 0.5 kN load increment, the deflection in the dial gauge of 0.01 mm accuracy is recorded manually. The laminate is closely observed to identify the first crack formation during the load application which is called as first crack load. After the first crack load formation, the loading process continues gradually till the laminate completely reaches its ultimate load. The load and deflection values were plotted to form a load deflection behavior graph. Energy absorption capacity is determined by measuring the area below the load deflection curve of the laminate. The last parameter ductility factor is determined by the ratio of deflection at ultimate load to deflection at first crack load. The above results were analyzed to assess the impact of filament thickness, type of warp knitted structure and number of layers in the laminate. Figure 5 represents the flexural test set up of ferrocement composites.

#### 2.1.4. Microstructure Analysis of Ferrocement Composites

The micro structural properties and elemental compositions of the PP filaments and PP warp knitted fabric reinforced ferrocement composites were determined by scanning electron microscope (SEM). The XL30 SEM instrument was used to investigate the morphological characteristics [41,42,43,44,45]. The scale range used in the SEM analysis was 5 µm with the resolution of 5000x. The samples were examined in the SEM instrument [46,47,48,49,50]. After the flexural strength test, mortar bonded with the fabrics was taken out from the ferrocement composites. Then, it was examined under SEM to understand the bonding between the mortar and fabrics.

## 3. Results and Discussion

### 3.1. Warp Knitted Fabric Characteristics

**Figure 6:**
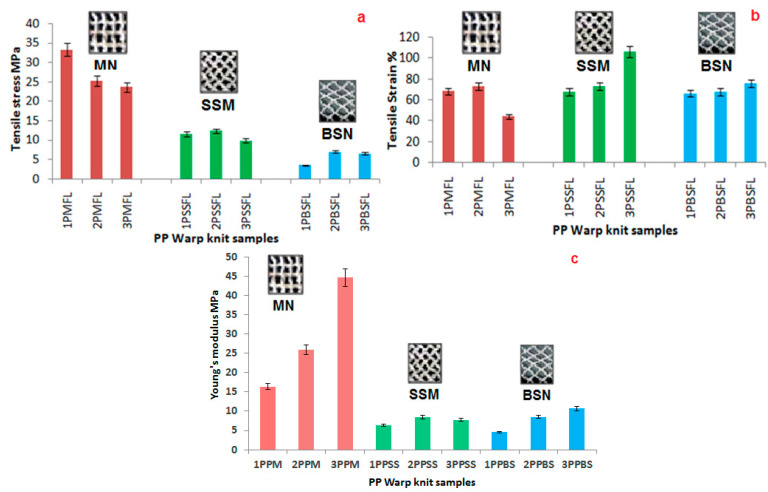
Tensile properties of Polypropylene warp knitted samples (**a**) tensile stress, (**b**) tensile strain, and (**c**) Young’s modulus.

Figure 6, represents the tensile stress, tensile strain and Young’s modulus of the polypropylene warp knitted samples respectively. In the Figure 6, 1PPM refers to the marquisette net fabric of 93 tex, 2PPM refers to the same structure produced with 187 tex and 3PPM refers to the same structure produced with 270 tex yarn count. Similarly, PPSS and PPBS refers to the warp knit fabric produced with small sandfly net and big sandfly net structure respectively and FL refers to the ferrocmeent laminate produced with the respective warp knit fabric.

Tensile test results show that marquisette net structure possess good tensile properties than the other two sandfly net structure. The reason is the vertical arrangement of yarns in the marquisette net structure. With respect to tensile strain, all the warp knitted fabric exhibit very good strain rate due to its nature of structure. Small mesh size sandfly net fabric has the second highest tensile strength due to the close arrangement of yarns in the mesh structure. Big mesh size sandfly net has lesser tensile properties due to the bigger mesh opening than the other two structures. The level of lapping movement in the warp knitted fabrics has an impact in the tensile properties [51].

### 3.2. First Crack Load Analysis

**Figure 7:**
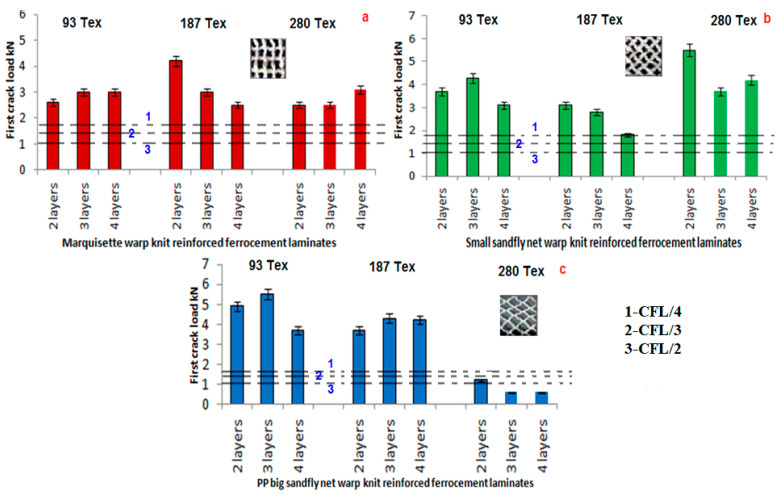
First crack load analysis of (**a**) marquisette, (**b**) small sandfly, and (**c**) big sandfly net polypropylene warp knitted reinforced ferrocement laminate.

Figure 7, represents the first crack load analysis of marquisette net, small sandfly net and big sandfly net warp knitted fabric reinforced ferrocement composites in comparison with the chicken mesh reinforced ferrocement composites.

The graphical representation clearly indicates that the warp knitted reinforced ferrocement composites performs better than chicken mesh ferrocement composites in the first crack load formation. The effect of filament denier, type of structure and number of layers in the laminate also analysed. The results show that sandfly net performs better than marquisette net structure. Three layer laminate performs better than two layer laminate and in many cases three layer laminate is better than four layer laminate. The filament denier has an effect in the first crack load in the sandfly small net structure. Among the two sandfly net, big mesh size performs better than the small mesh size. As the filament denier increases to an extent, the flexural properties increases and at the same time higher filament denier results in lower bonding strength between warp knitted fabric and cement mortar which affects the flexural properties [52].

### 3.3. Ultimate Load Analysis

**Figure 8:**
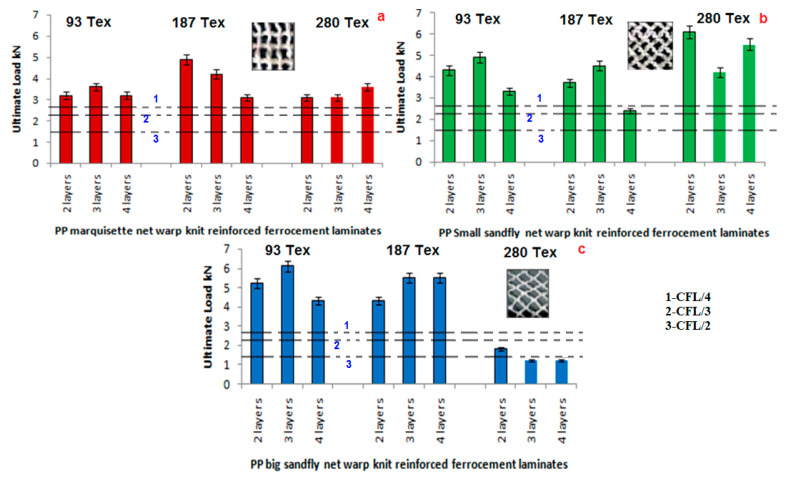
Ultimate load analysis of (**a**) marquisette, (**b**) small sandfly, and (**c**) big sandfly net polypropylene warp knitted reinforced ferrocement composites.

Figure 8, represents the ultimate load analysis of marquisette net, small sandfly net, big sandfly net warp knitted fabric reinforced ferrocement composites in comparison with the chicken mesh reinforced ferrocement composites.

The graphical representation clearly indicates that the ultimate load of warp knitted reinforced ferrocement composites is higher than chicken mesh ferrocement composites. With respect to the effect of filament denier, type of structure and number of layers in the laminate, the results show that sandfly net performs better than marquisette net structure. Three layer laminate is superior to two layer laminate and four layer composites. The filament denier shows a positive impact in the ultimate load of the warp knitted reinforced ferrocement composites which were reduced due to the increase in the number of layers [52].

### 3.4. Load Deflection Behavior

**Figure 9:**
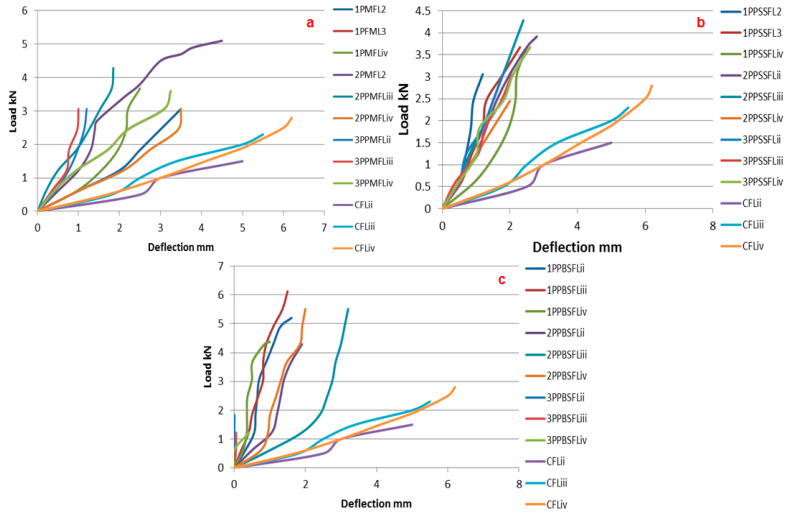
Load deflection behaviour of (**a**) marquisette, (**b**) small sandfly, and (**c**) big sandfly net polypropylene warp knitted reinforced ferrocement composites.

Figure 9, represents the load deflection behaviour of marquisette net, sandfly small size net mesh and sandfly big size mesh warp knitted fabric reinforced ferrocement composites in comparison with the chicken mesh reinforced ferrocement composites.

The load-deflection behaviour graph shows that warp knitted fabric reinforced ferrocement laminate shows higher breaking load with lesser elongation in comparison with the chicken mesh reinforced laminate which shows lesser load with higher elongation. This is due to the thin cross section of the chicken mesh wire compared to the warp knitted fabrics.

**Figure 10:**
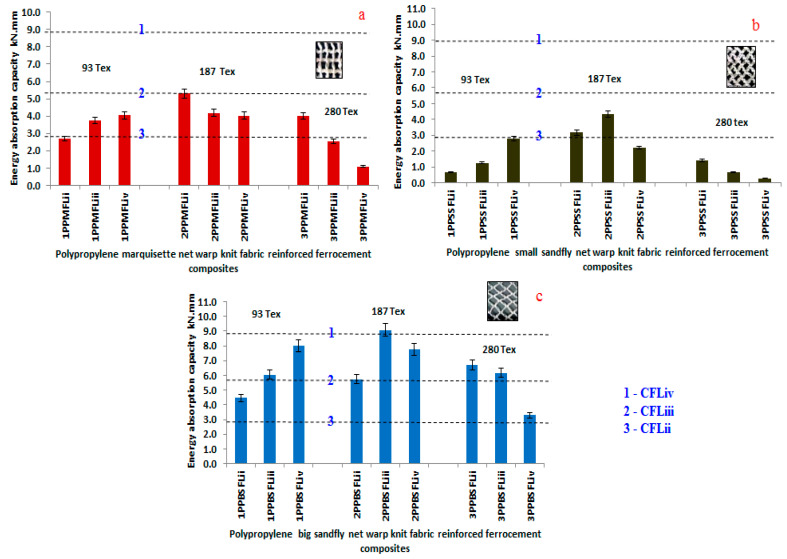
Energy absorption capacity of (**a**) marquisette, (**b**) small sandfly, and (**c**) big sandfly net polypropylene warp knitted reinforced ferrocement composites.

Figure 10, represents the energy absorption factor analysis of marquisette net, small sandfly net, big sandfly net warp knitted fabric reinforced ferrocement composites in comparison with the chicken mesh reinforced ferrocement composites.

**Figure 11:**
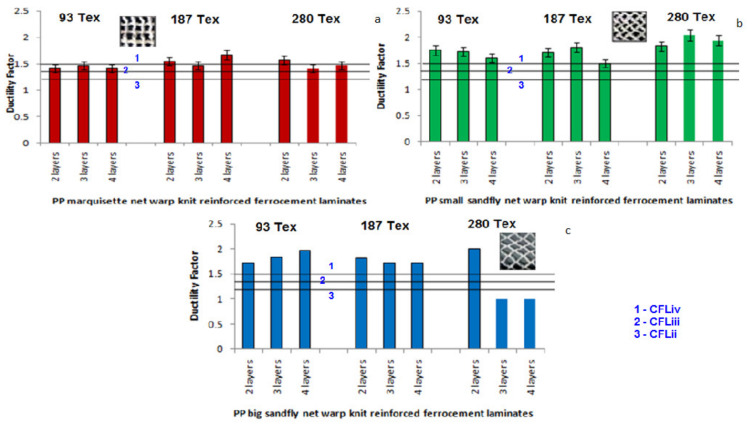
Ductility factor analysis of (**a**) marquisette, (**b**) small sandfly, and (**c**) big sandfly net polypropylene warp knitted reinforced ferrocement composites.

The graphical representation indicates that energy absorption factor of warp knitted reinforced ferrocement composites is higher than chicken mesh ferrocement composites. Further analysis shows that marquisette net structure and small sandfly net structure possess high energy absorption capacity than other composites. Big sandfly net shows lesser energy absorption factor in its composites. The filament denier doesn’t show any impact in the energy absorption factor, but the number of layers has a positive impact in the results. Three layers in the laminate possess good energy absorption factor than other composites. Figure 11, represents the ductility factor analysis of marquisette net, small sandfly net, big sandfly net warp knitted fabric reinforced ferrocement composites in comparison with the chicken mesh reinforced ferrocement composites. Among all the composite samples analysed, where the 2-PP/MFL/2 shows the maximum energy absorption capacity value.

The graphical representation indicates that there is an improvement in ductility factor due to warp knitted fabrics compared to chicken mesh reinforcement. In this experiment, there is no significant difference in the energy absorption factor and ductility factor and the reason is the thin cross section chicken mesh wire and the higher elongation of conventional ferrocement composites. The above analysis helps to prove that the replacement of conventional chicken mesh wire with the textile structure preferably warp knitted made up of synthetic filament yarns which improved the flexural properties of ferrocement composites [52].

### 3.5. Micro Structure Analysis

**Figure 12:**
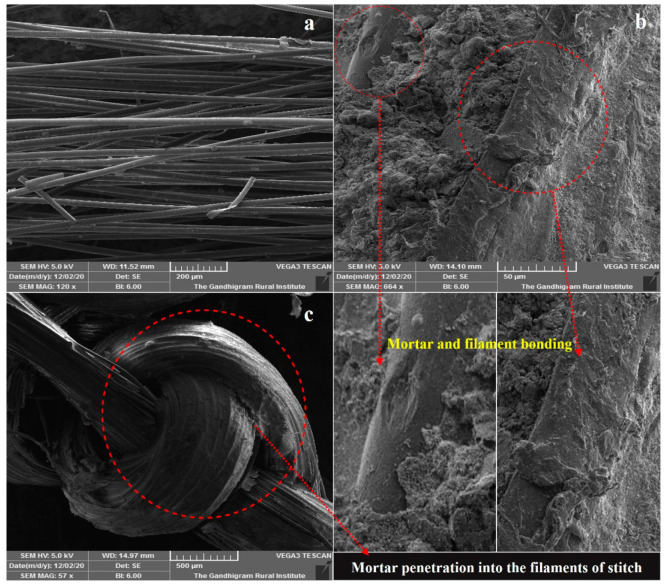
SEM images of (**a**) polypropylene filaments, (**b**) warp knitted fabric reinforced ferrocement composites, and (**c**) mortar penetration into the warp knitted fabric stitch.

Figure 12 represents the SEM images of PP filaments, ferrocement and warp knitted fabric in composites respectively. Figure 12a exhibits the smooth surface of PP filaments with small projections in the outer layer.

In Figure 12b, the bonding is clearly visible between the mortar mix and warp knitted fabrics. In Figure 12c, the penetration of mortar mix inside the warp knitted stitches of the filaments is clearly visible. As the curing days increases, the bond between mortar mix and warp knitted fabrics were increased due to the growth of microstructure. Thick mortar particles get patched to the fabric stitches leads to increase in strength. In big sandfly ferrocement composites, the mortar get tightly fixed between the diagonal strips postponed the first crack formation in flexure.

### 3.6. Statistical Data Analysis

Flexural properties of polypropylene warp knitted reinforced ferrocement composites were evaluated with statistical analysis of variance at 95% significance level with SAS system (version 8 for windows) for estimating the significance level of experimental variables on the flexural properties of the ferrocement composites. The *p*-values were calculated to analyze whether there is a significant improvement in the flexural properties with the polypropylene warp knitted reinforcement. The statistical values are given in Table 6, Table 7 and Table 8. It is evident that there is a significant difference is there in the first crack load and ultimate load between the control sample and pp reinforced ferrocement composites. Also it is noted that the energy absorption factor and ductility factor doesn’t show any significant difference in the statistical analysis. This is due to the lesser deflection of polypropylene warp knitted reinforced ferrocement composites which are already proved previously in an experiment [52].

## 4. Conclusions

The above segment gives a detailed analysis of flexural properties of warp knitted fabric reinforced ferrocement composites against the regular chicken mesh reinforced ferrocement composites. From the above analysis, the following conclusions were made.

- Among the three warp knitted fabrics, marquisette net fabric possesses higher tensile properties than the sandfly net fabrics due to straight alignment of yarns in the fabrics. Also, the warp knitted fabrics possess good tensile strain properties due to the nature of its structure. For the 93 tex samples, big sandfly net has the highest tensile strain value of 98.4%, which is 61% higher than the 93 tex PP marquisette and 22% higher than 93 tex PP small sandfly warp knitted fabrics. Furthermore, small sandfly net exhibit higher tensile strain than the marquisette structure due to the geometry of filament arrangement in the fabric.
- First crack load of sandfly structure is better than the marquisette structure. The reason is due to the diagonal positioning of yarns in the structure. Among the two sandfly net, big mesh has better first crack load than the small sandfly net due to better cement mortar penetration. The same phenomenon reflects in the ultimate load of the composites also.
- Load deflection behaviour of the composites clearly indicates the higher breaking load of the warp knitted fabric reinforced ferrocement composites than the chicken mesh reinforced ferrocement composites.
- Marquisette fabric and small sandfly net composites possess high energy absorption factor than the other composites due to higher deflection in the composites for the given load. The reason is due to the lesser cement fabric bonding.
- All the warp knitted reinforced ferrocement composites has improved ductility factor compared to conventional chicken mesh reinforced ferrocement composites When using chicken mesh the corrosion factor is the main drawback, so replacing it with warp knitted fabrics enhances corrosion resistance.
- The filament denier has a positive impact in the flexural properties. With respect to number of layers, three layers in the laminate shows improved performance properties than the 2- and 4- layer composites.
- Microstructure analysis on ferrocement confirms the bonding between the mortar mix and warp knitted fabrics and the penetration of mortar mix inside the warp knitted stitches of the filaments is clearly visible. In big sandfly ferrocement composites, the mortar get tightly fixed between the diagonal strips postponed the first crack formation in flexure.

It can be concluded that sandfly net warp knitted structure with bigger mesh size and moderate filament denier with three layers in the laminate will be an effective replacement for the chicken mesh wire in the ferrocement composites in terms of flexural properties.

## Figures and Tables

**Table 1:** Physical properties of OPC cement (53 grade).

Property	Value
Consistency	36%
Intial setting time	40 min
Final setting time	600 min
Specific gravity	3.15

**Table 2:** Physical properties of fine aggregate.

Property	Value
Specific gravity	2.63(no unit)
Grading zone	II
Water absorption	1%
Fineness modulus	2.6 (no unit)
Bulk density (a) Loose (b) Compacted	1530 kg/m^3^ 1630 kg/m^3^

**Table 3:** Specifications of chicken mesh wire.

Property	Value
Raw material	Galvanized Iron
Mesh shape	Hexagonal
Diameter of wire, mm	0.71 mm
Weight g/sq.m	390
Density	7820 kg/m^3^

**Table 4:** Details of the 27 Ferrocement laminate samples.

Sample Code	Structure and Mesh Size	Sample Code	Structure and Mesh Size	Sample Code	Structure and Mesh Size	Filament Denier	No. of Layers
1-PP/MFL/2	Marquisette net structure 5 × 5 mm mesh size	1-PP/SSFL/2	Sandfly small net structure 5 mm × 5 mm mesh size	1-PP/BSFL/2	Sandfly big net structure 10 mm × 10 mm mesh size	93 tex	2
1-PP/MFL/3		1-PP/SSFL/3		1-PP/BSFL/3			3
1-PP/MFL/4		1-PP/SSFL/4		1-PP/BSFL/4			4
2-PP/MFL/2		2-PP/SSFL/2		2-PP/BSFL/2		187 tex	2
2-PP/MFL/3		2-PP/SSFL/3		2-PP/BSFL/3			3
2-PP/MFL/4		2-PP/SSFL/4		2-PP/BSFL/4			4
3-PP/MFL/2		3-PP/SSFL/2		3-PP/BSFL/2		280 tex	2
3-PP/MFL/3		3-PP/SSFL/3		3-PP/BSFL/3			3
3-PP/MFL/4		3-PP/SSFL/4		3-PP/BSFL/4			4

**Table 5:** Details of chicken mesh reinforced Ferrocement composites.

Sample Code	Sample Description
CFL/2	2 layer chicken mesh reinforced ferrocement laminate
CFL/3	3 layer chicken mesh reinforced ferrocement laminate
CFL/4	4 layer chicken mesh reinforced ferrocement laminate

**Table 6:** Analysis of variance between chicken mesh ferrocement and warp knitted structure reinforced ferrocement.

Variance Analysis	Degrees of Freedom (df)	Sum of Square Value (s)	Mean Square Value (ms)	F-Value	*p*-Value
First crack load kN					
Marquisette net structure	3	5.446667	1.815556	4.431186	0.057566 ^a^
Small sandfly net structure	3	9.146667	3.048889	22.4918	0.001154 ^a^
Big sandfly net structure	3	24.52917	8.176389	24.36672	0.000927 ^a^
Ultimate load kN					
Marquisette net structure	3	5.316667	1.772222	3.883141	0.074112 ^a^
Small sandfly net structure	3	6.633333	2.211111	4.769323	0.049742 ^a^
Big sandfly net structure	3	22.9425	7.6475	22.22034	0.001193 ^a^
Energy absorption factor kN.mm					
Marquisette net structure	3	3.853333	1.284444	0.340375	0.797495
Small sandfly net structure	3	12.17	4.056667	0.838299	0.520392
Big sandfly net structure	3	15.35	5.116667	3.459155	0.091495
Ductility factor					
Marquisette net structure	3	0.046158	0.015386	2.114122	0.199799
Small sandfly net structure	3	0.432967	0.144322	11.23373	0.007109
Big sandfly net structure	3	0.562558	0.187519	1.82274	0.243383

Note: ^a^ significant for α = 0.05.

**Table 7:** Analysis of variance by the filament denier between marquisette, small sandfly and big sandfly structure reinforced ferrocement composites.

Variance Analysis	Degrees of Freedom (df)	Sum of Square Value (s)	Mean Square Value (ms)	F-Value	*p*-Value
First crack load kN					
Between marquisette and small sandfly	1	0.067222	0.067222	0.183681	0.679535
Between small sandfly and big sandfly	1	5.445	5.445	4.229126	0.073762 ^a^
Between marquisette and big sandfly	1	6.722222	6.722222	5.591497	0.045627 ^a^
Ultimate load kN					
Between marquisette and small sandfly	1	0.035556	0.035556	0.089888	0.771962
Between small sandfly and big sandfly	1	6.242222	6.242222	7.557424	0.025095
Between marquisette and big sandfly			5.335556	5.312682	0.050084 ^a^
Energy absorption factor kN. mm ^a^					
Between marquisette and small sandfly	1	1.62	1.62	0.698276	0.427614
Between small sandfly and big sandfly	1	0.680556	0.680556	0.135858	0.721997
Between marquisette and big sandfly		4.400556	4.400556	0.697194	0.427958
Ductility factor					
Between marquisette and small sandfly	1	0.0002	0.000200	0.016495	0.900977
Between small sandfly and big sandfly	1	0.025689	0.025689	0.316701	0.589015
Between marquisette and big sandfly	1	0.021356	0.021356	0.177955	0.684244

Note: ^a^ significant for α = 0.05.

**Table 8:** Analysis of variance by the number of layers in the reinforcement between marquisette, small sandfly and big sandfly structure reinforced ferrocement composites.

Variance Analysis	Degrees of Freedom (df)	Sum of Square Value (s)	Mean Square Value (ms)	F-Value	*p*-Value
First crack load kN					
Between 2 layer and 3 layer	1	0.98	0.98	2.473186	0.053654 ^a^
Between 3 layer and 4 layer	1	1.027222	1.027222	5.111265	0.154448
Between 2 layer and 4 layer	1	0.000556	0.000556	0.965974	0.001937 ^a^
Ultimate load kN					
Between 2 layer and 3 layer	1	1.933889	1.933889	7.888952	0.062888 ^a^
Between 3 layer and 4 layer	1	2.067222	2.067222	6.542418	0.033762
Between 2 layer and 4 layer		0.002222	0.002222	0.953625	0.0036 ^a^
Energy absorption factor kN.mm					
Between 2 layer and 3 layer	1	2.347222	2.347222	2.017429	0.19328
Between 3 layer and 4 layer	1	0.005	0.005	0.00099	0.975666
Between 2 layer and 4 layer		2.89	2.89	0.656818	0.444361
Ductility factor					
Between 2 layer and 3 layer	1	0.043022	0.043022	0.668985	0.437087
Between 3 layer and 4 layer	1	0.00245	0.00245	0.221219	0.650669
Between 2 layer and 4 layer	1	0.066006	0.066006	1.016752	0.342812

Note: ^a^ significant for α = 0.05.

## Data Availability

Not applicable.
